# A Q Methodological Study on Algorithmic Intimacy, Social Connection, Well-Being, and the Boundaries of Autonomy in University Students’ Conversational Artificial Intelligence Experiences

**DOI:** 10.3390/bs16071258

**Published:** 2026-07-22

**Authors:** Kyoung Yeon Moon, Taeeun Shim

**Affiliations:** 1 Dharma College, Dongguk University, Jung-gu, Seoul 04620, Republic of Korea; kymoon1231@dongguk.edu; 2 Bright College, Hankyong National University, Anseong-si 17579, Gyeonggi-do, Republic of Korea

**Keywords:** algorithmic intimacy, autonomy, conversational AI, Q methodology, social connection, well-being

## Abstract

This study explores how university students experience conversational artificial intelligence (AI) in the era of generative AI from the perspectives of algorithmic intimacy, social connection, well-being, and autonomy, and identifies their subjective meaning structures. Specifically, it examines how university students perceive conversational AI in relation to emotional regulation, self-understanding, relational boundaries, and autonomy, and how these perceptions relate to intimacy and well-being. Unlike previous studies focused primarily on AI use and acceptance, this study analyses how students interpret their experiences with conversational AI and negotiate relational boundaries. Using Q methodology, we developed 40 Q statements from literature review and in-depth interviews. Fifty-six university students in South Korea with experience using conversational AI completed the Q-sorting process. A four-factor solution explained 55% of the total variance and yielded four distinct types: (1) Relationship-Negating Instrumentalists, (2) Selective Emotional Utilizers, (3) Reflective Distancers, and (4) Autonomy Defenders. Although none of the groups viewed AI as a complete substitute for human relationships, they differed in their acceptance of algorithmic intimacy, use of AI for emotional support and regulation, concerns about dependence, and efforts to protect autonomy. These findings suggest that conversational AI experiences involve negotiating intimacy and boundaries rather than producing uniformly positive or negative outcomes. This study complements variable-centred approaches by identifying distinct subjective meaning structures in university students’ human–AI relationship experiences.

## 1. Introduction

### 1.1. Conversational AI, Algorithmic Intimacy, and Relational Boundaries

The rapid diffusion of generative artificial intelligence (GenAI), particularly large language model (LLM)-based conversational agents, has reshaped the ways in which users interact with digital technologies in educational, informational, emotional, and relational contexts. In higher education, recent studies show that university students increasingly perceive GenAI as useful for learning, idea generation, personalised feedback, writing support, and academic problem-solving, while also expressing concerns about accuracy, dependence, authorship, privacy, and ethical use (Chan & Hu, 2023; Dwivedi et al., 2023; Kasneci et al., 2023). At the same time, conversational AI is no longer limited to information retrieval or task execution. Its immediate, personalised, and seemingly empathic responses have made it increasingly relevant to self-disclosure, emotional support, loneliness and anxiety management, and social connection (Merrill et al., 2025; Papneja & Yadav, 2025; Volpato et al., 2025).

Conversational AI is increasingly understood not merely as a technical interface but as an interactive environment in which users may experience responsiveness, social presence, emotional validation, and perceived understanding. Because users employ conversational AI to express emotions, disclose concerns, seek advice, and reflect on choices, such interactions may become part of how they understand themselves, set relational boundaries, and perceive their autonomy. Consequently, conversational AI raises broader questions about how users construct intimacy, trust, reliance, and distancing with non-human agents.

These developments position conversational AI as a relational object within users’ subjective experiences (Nass & Moon, 2000; Gnewuch et al., 2017). Scholars have used concepts such as “AI-mediated intimacy” and “algorithmic intimacy” to explain how users attribute social qualities such as understanding, empathy, intentionality, and care to AI and may develop a sense of emotional intimacy through repeated interactions (Bickmore & Picard, 2005; Turkle, 2017; Elliott, 2022). The key issue is not whether AI possesses genuine emotions or intentions, but how users interpret AI-generated responses as relational signals and construct intimacy and distancing through those responses.

In this study, algorithmic intimacy does not refer to emotions or intentions within AI but to the process through which users may experience personalised responses, immediate feedback, non-judgemental dialogue, and repeated interactions as forms of understanding, comfort, and emotional closeness. This conceptualisation builds on research showing that people respond socially to computers and media, as well as on studies of relational agents designed to engage users in socio-emotional interaction. These interactional affordances may lead users to experience conversational AI as a social companion or an emotional conversational partner (Reeves & Nass, 1996; Bickmore & Picard, 2005). This concept extends research showing that people respond socially to computers and media, as well as studies of relational agents that emotionally interact with users. Because conversational AI responds immediately, avoids negative evaluation, and provides personalised advice and empathetic language, users may experience it as a social companion or an emotional conversational partner (Gnewuch et al., 2017; Ta et al., 2020).

AI-mediated intimacy also relates to parasocial relationship theory (Liebers & Schramm, 2019). Parasocial relationships describe how individuals develop one-sided feelings of intimacy and attachment toward media figures or virtual entities despite limited reciprocal interaction (Horton & Wohl, 1956; Tukachinsky, 2011). Recent research has extended this concept to chatbots, virtual characters, and conversational AI (Hoffner & Bond, 2022). Although conversational AI does not provide the reciprocity of human relationships, users may still develop feelings of intimacy and trust, patterns of repeated reliance, and emotional expectations through continued interaction (Ta et al., 2020). Therefore, researchers should move beyond asking whether AI relationships are “real” and instead consider how users interpret and experience AI. This perspective highlights the relational meanings users assign to AI and the boundaries they establish, rather than reducing conversational AI merely to a substitute for human relationships or a functional tool.

Attachment theory also helps explain conversational AI experiences. The theory describes people’s tendency to seek sources of security and emotional support during periods of anxiety, loneliness, or stress (Mikulincer & Shaver, 2016). Conversational AI often serves this function by responding immediately, avoiding judgement, and remaining available at any time, which can lead users to perceive it as an emotionally supportive conversational partner (Fitzpatrick et al., 2017; Ta et al., 2020). For university students, experiences such as sharing concerns with AI that they hesitate to discuss with others or turning to AI first during difficult situations extend beyond simple technology use. These behaviours reflect emotional regulation and the pursuit of psychological stability (Ho et al., 2018; Lucas et al., 2014).

In some cases, conversational AI may function as a psychological “secure base”, highlighting the need to analyse human–AI relationships through the lenses of emotional dependence and self-regulation (Hoffner & Bond, 2022). However, users do not form actual reciprocal relationships with AI. Instead, they interpret AI’s responsiveness in relational terms and use it to meet emotional needs. Therefore, this study treats attachment as a bounded concept that explains how users employ conversational AI for emotional self-disclosure, emotional processing, and anxiety alleviation rather than as a direct equivalent of interpersonal attachment.

### 1.2. Social Connection, Well-Being, and Autonomy in Conversational AI Use

However, intimacy with conversational AI remains an inherently ambivalent phenomenon that cannot be explained solely in terms of positive relationship formation (Dwivedi et al., 2023). Interactions with AI may reduce loneliness, facilitate emotional processing, encourage self-disclosure, promote emotional stability, and supplement social support. However, they can also foster avoidance of interpersonal relationships, emotional dependence, feelings of emptiness, weakened autonomy, reduced independent thinking, and scepticism about the authenticity of the relationship (Kasneci et al., 2023; Ta et al., 2020; Ho et al., 2018). While users may appreciate the convenience of receiving advice, decision support, and emotional responses from conversational AI, they may also worry that AI increasingly shapes or replaces their own individual thinking and choices.

Consequently, conversational AI use involves not only emotional intimacy but also agency, self-determination, and cognitive autonomy in complex ways. Users may recognise that AI’s apparent “understanding” reflects algorithmic processing rather than genuine empathy, even as they feel understood by it. At the same time, they may acknowledge that the comfort they derive from AI conversations partly rests on an illusion of genuine understanding and empathy (Epley et al., 2007). Therefore, conversational AI experiences reflect a complex tension between intimacy and distance, comfort and anxiety, efficiency and dependence, and social connection and potential isolation (Bond et al., 2020). This ambivalence motivates the present study to examine differences in users’ subjective meaning structures rather than reducing conversational AI experiences to a single positive or negative effect.

This ambivalence becomes even more important when viewed through the lenses of social connection and well-being (Dwivedi et al., 2023). Social connectedness reflects a person’s sense of belonging to others and the broader social world, whereas well-being encompasses emotional stability, life satisfaction, and psychological functioning (R. M. Lee & Robbins, 1995; Ryff, 1989). Conversational AI may support social connectedness and well-being by facilitating self-expression and alleviating loneliness. However, it may also undermine well-being by displacing human relationships or encouraging avoidance of real-world social interactions (Ta et al., 2020; Nowland et al., 2018). In this study, well-being is examined through users’ subjective experiences of emotional stability, comfort, emptiness, anxiety, self-regulation, and concerns about dependence. Rather than measuring the objective effects of conversational AI use on mental health, we focus on how users interpret well-being-related experiences within interactions with conversational AI. As these interactions accumulate, users may experience AI as a familiar conversational partner while also recognising its relational limitations and experiencing emotional emptiness or anxiety about dependence (Bickmore & Picard, 2005). Consequently, users construct intimacy with AI in diverse ways (Bond et al., 2020). For some, conversational AI can serve as a valuable resource for social connection; for others, it raises concerns about emotional substitution, avoidance of human relationships, or infringement of autonomy.

Conversational AI experiences may also intersect with processes of self-understanding and autonomy development in young adulthood. In this context, identity concerns how young people negotiate agency, relational boundaries, and self-determination in their interactions with algorithmic systems. When university students seek advice from AI, reflect on their emotions, or rely on AI for writing and decision-making support, they often reconsider where “my thoughts,” “my judgments,” and “my expressions” begin and end. In this process, conversational AI can facilitate self-reflection and expression, but it can also create concerns about excessive reliance on AI-generated responses. Accordingly, AI use can be understood as a context in which young people negotiate self-boundaries, autonomy, and relational identity within a digital environment. Rather than determining identity directly, conversational AI serves as a mediating environment that reveals ongoing negotiations over self-understanding, relational judgment, emotional dependence, and distancing.

### 1.3. Korean University Students in the Generative AI Context

The Korean context warrants particular attention. South Korea’s advanced digital infrastructure and rapid adoption of new technologies have facilitated the integration of generative AI into everyday life. University students increasingly use AI for academic work, career preparation, self-expression, emotional regulation, and relationship advice (Organisation for Economic Co-Operation and Development [OECD], 2025; Jun, 2024). At the same time, many Korean university students face intense academic competition, employment uncertainty, relationship fatigue, evaluation pressure, and emotional isolation (Y. S. Yang, 2026; H. Lee et al., 2026). These conditions may increase the likelihood that conversational AI is used not only as a learning aid but also as a source of emotional support and social connection (Fitzpatrick et al., 2017; Ta et al., 2020).

In this context, Korean university students’ interactions with conversational AI may likely to reflect not only individual preferences but also broader social pressures surrounding academic achievement, career preparation, emotional self-management, and relational fatigue. These conditions make the Korean case especially useful for examining how students negotiate the boundaries between efficiency and dependence, emotional support and relational substitution, and AI-assisted self-reflection and the protection of autonomy.

However, Korean higher education remains in a transitional phase, with evaluation standards, ethical norms, and institutional boundaries for AI use still evolving (Korean Council for University Education, 2025). Against this backdrop, Korean university students may experience AI as a complex phenomenon in which efficiency and anxiety, convenience and dependence, intimacy and distancing, and academic support and normative tension coexist (Kasneci et al., 2023). Analysing these experiences therefore offers a contextually situated case for understanding how young people in the generative AI era negotiate boundaries among technological assistance, emotional support, relational engagement, and autonomy.

### 1.4. Research Gap, Methodological Approach, and Research Questions

Despite growing interest in AI, a substantial body of existing research adopts variable-centred analyses that focus on AI-use frequency, technology acceptance, perceived usefulness, trust, usage intention, and academic performance (Zawacki-Richter et al., 2019). Although these approaches explain general trends in AI use and relationships among variables, they provide limited insight into how users construct meaning around their relationships with AI or how algorithmic intimacy, social connection, well-being, and autonomy are configured within distinct subjective meaning structures. Some users experience intimacy with AI as a source of comfort and self-expression, whereas others view it as a source of dependence and anxiety or as a useful tool that requires emotional distance. Therefore, there is a need to identify and analyse differences in university students’ subjective perceptions rather than reduce conversational AI experiences to a single average tendency.

Accordingly, this study shifts attention away from measuring the frequency or effects of conversational AI use and examines how university students interpret, justify, limit, and regulate their engagement with AI. Specifically, it investigates how students position conversational AI within meaning structures involving algorithmic intimacy, social connection, well-being, and autonomy. By analysing how students construct conversational AI as a relational object, this study provides subjectivity-centred empirical evidence for discussions of human–AI relationships in the era of generative AI.

To identify shared subjective viewpoints in university students’ experiences with conversational AI, this study applies Q methodology. Q methodology enables researchers to compare subjective perception structures and identify configurations of views shared across participants (Brown, 1980; Watts & Stenner, 2012). Complex socio-psychological phenomena, such as algorithmic intimacy, social connection, well-being, and autonomy, may not be fully captured by existing scales alone.

By asking participants to sort statements according to their relative agreement or disagreement, Q methodology reveals which viewpoints they endorse, reject, or hold in tension. Accordingly, this approach aligns with the study’s objective of examining how university students construct subjective meanings around intimacy, social connection, well-being, and autonomy in their interactions with conversational AI.

The research questions (RQs) are as follows:

RQ1. How do university students make sense of algorithmic intimacy, social connection, well-being, and autonomy in their interactions with conversational AI?
RQ2. What distinct subjective types can be identified, and how does each type combine perceived utility, emotional use, relational boundary-setting, and concerns about dependence?
RQ3. What do these types suggest for understanding university students’ human–AI relationships and for informing educational and ethical discussions concerning social connection, well-being, and the protection of autonomy?

## 2. Materials and Methods

Q methodology systematically explores individuals’ subjective perception structures by integrating qualitative inquiry with quantitative analysis. Researchers use Q-sorting to examine participants’ subjective judgments, interpretations, and priorities regarding a specific phenomenon and to identify types that share similar patterns of perception (Kil et al., 2020; McKeown & Thomas, 2013). Because Q methodology focuses on differences in subjective meaning structures rather than averaging responses across variables, it aligns closely with this study’s objective of understanding how participants construct and interpret conversational AI experiences.

We adopted Q methodology because usage frequency and perceived usefulness alone cannot fully capture the complexity of conversational AI experiences. University students may view conversational AI as a learning tool, an emotional conversational partner, a channel for self-disclosure, a source of social connection, or a technical system that poses risks of dependence and diminished autonomy. Rather than testing the average effects of conversational AI use, this study examines how participants configure algorithmic intimacy, social connection, well-being, and autonomy within their subjective experiences.

Q-methodological research generally involves constructing a Q concourse, selecting a final Q sample, recruiting a P sample, conducting Q sorts, and identifying shared subjective viewpoints through factor analysis (Brown et al., 1999). Accordingly, this study followed four stages: (1) construction and refinement of the Q concourse and Q sample, (2) recruitment of participants with conversational AI experience, (3) Q sorting and post-sort comments, and (4) factor extraction and interpretation using KenQ Analysis Desktop Edition (KADE). Detailed procedures for Q-sample development, participant recruitment, Q sorting, post-sort comments, and factor analysis are presented in the following subsections.

Factor interpretation drew on factor-specific Z-scores, distinguishing statements, consensus statements, extreme-ranking patterns, and participants’ post-sort explanations. The procedures used for data processing, factor extraction, and factor interpretation are outlined in Section 2.3.

To meet research ethics requirements, we obtained written informed consent from all participants after explaining the study’s purpose, procedures, anonymity protections, exclusive research use of the data, and the right to withdraw from the study at any time. We anonymised all personal information before analysis to protect participant confidentiality. The participating institutions were also anonymised in the manuscript. The Institutional Review Board approved this study (DUIRB2026-01-01).

### 2.1. Q Concourse and Q Sample

In Q methodology, the Q concourse comprises the full range of socially circulating discourses, experiential interpretations, and emotional expressions related to the research topic. To capture diverse meaning structures surrounding university students’ experiences of algorithmic intimacy, social connection, well-being, and autonomy in relation to conversational AI, we constructed the concourse through a combination of a literature review and in-depth interviews.

First, we reviewed studies on algorithmic intimacy, the emotional dynamics of human–AI relationships, AI companion use and well-being, relational authenticity, AI ethics, and related social tensions. Consistent with the theoretical framework presented in the previous section, we focused on algorithmic intimacy, parasocial relationships, attachment and emotional regulation, self-disclosure, social connection, well-being, the complementarity or substitution of human relationships, and issues of AI ethics and autonomy (Chu et al., 2025; Hong et al., 2025; Zhang et al., 2025). Based on this review, we developed 46 preliminary statements. These statements captured perceptions of conversational AI as a functional tool and as a source of emotional support, relational intimacy, and social connection, as well as concerns about dependence, and the potential infringement of autonomy.

Next, we conducted semi-structured interviews with 10 university students from five universities who had experience using conversational AI. The sample included six female and four male students from diverse academic disciplines to ensure a broad range of perspectives. We asked participants questions such as: “What role does AI play for you?”, “Have you ever felt a sense of intimacy in your interactions with AI? If so, when?”, “Are there things that you find difficult to tell other people but share with AI?”, and “How does your decision to share or withhold such information relate to concerns about evaluation, stigma, burden, or interpersonal repercussions?” These questions explored whether participants viewed conversational AI as an informational tool, an emotional support resource, a complement to human relationships, or a technical system from which emotional distance was needed. After transcribing and analysing the interview data, we derived 65 additional statements.

We combined the 46 literature-based statements and the 65 interview-based statements to create an initial concourse of 111 statements. The reduction in the concourse from 111 to 40 statements was conducted through a structured, criteria-based refinement process. First, the statements were screened for semantic redundancy, repetitive wording, compound meanings, and conceptual overlap. Statements expressing nearly identical meanings were removed or merged, whereas conceptually adjacent statements were retained when they captured distinguishable experiential aspects, such as emotional attachment, habitual use, perceived dependence, relational substitution, and the protection of autonomy.

Second, two researchers and one Q-methodology expert independently reviewed the remaining statements for clarity, single-meaning construction, self-referential wording, theoretical relevance, and the absence of leading or value-laden phrasing. The reviewers also examined whether each statement could be evaluated by participants on the basis of their own experiences with conversational AI. Differences in wording or category assignment were resolved through discussion and consensus. Because the review process was intended as a qualitative refinement of the Q sample rather than a quantitative rating exercise, no formal inter-rater reliability coefficient was calculated.

Third, the statements were examined for conceptual coverage. The final Q sample was designed to represent the following theoretical categories: algorithmic intimacy; attachment formation and attachment anxiety; relational complementarity and substitution; well-being and emotional regulation; relational authenticity; identity-related uncertainty and autonomy; functional utility; social influence; concerns about reduced independent thinking; perceptions of fairness; and ethical and social tensions. These categories were used as conceptual coverage checks rather than as fixed numerical quotas.

Fourth, the researchers assessed the balance of the final set across four broader analytic dimensions—algorithmic intimacy, social connection, well-being and emotional regulation, and boundaries of autonomy—and across positive or acceptance-oriented, negative or concern-oriented, and ambivalent or boundary-setting positions. This procedure was intended to prevent the Q sample from privileging a single theoretical or evaluative orientation.

The final Q sample consisted of 40 statements. Each statement was assigned a primary analytic dimension for reporting purposes, although some statements could conceptually relate to more than one dimension. Of these, 12 primarily addressed algorithmic intimacy, 8 addressed social connection, 10 addressed well-being and emotional regulation, and 10 addressed boundaries of autonomy. In terms of valence, 15 statements represented positive or acceptance-oriented positions, 18 represented negative or concern-oriented positions, and 7 represented ambivalent or boundary-setting positions. Ambivalent and boundary-setting statements were retained because the purpose of Q methodology is to capture nuanced configurations of subjective meaning rather than to reduce participants’ experiences to simple approval or disapproval. Table 1 presents the final statements, their primary analytic dimensions, and their valence classifications.

This configuration reflects our view that algorithmic intimacy extends beyond a simple emotional experience and instead represents a multi-layered meaning structure involving attachment, well-being, cognitive autonomy, social norms, and ethical judgements. The research team judged that the final 40 statements provided adequate conceptual coverage and theoretical balance for examining the range of university students’ AI-mediated experiences addressed in this study.

### 2.2. Participants: P Sample

The P sample comprises the participants who complete the Q-sorting process and express their subjective viewpoints through the arrangement of statements. Q methodology does not seek to generalise findings to a broader population; instead, it aims to identify and explore diverse perspectives in depth. Therefore, researchers generally use relatively small samples (Watts & Stenner, 2012). The adequacy of a P sample is determined primarily by whether it captures a sufficiently diverse range of perspectives relevant to the research topic, rather than by conventional statistical power requirements. Because Q methodology focuses on identifying shared patterns of subjective thought across participants’ Q sorts rather than generalising results, researchers prioritise diversity of viewpoints over sample size (Y. Yang, 2016). Accordingly, this study prioritised capturing a wide range of subjective perspectives on conversational AI experiences rather than achieving statistical representativeness.

We selected university students with conversational AI experience as the P sample. To participate, students had to meet four criteria: (1) they had to be currently enrolled in a university, (2) they had to have experience using conversational AI, including ChatGPT developed by OpenAI, although specific model versions were not recorded, (3) they had to be able to understand the Q-sorting instructions and complete the sorting procedure independently, and (4) they had to provide voluntary consent to participate in the study.

We ultimately recruited 56 university students. As Table 2 shows, the sample was relatively balanced by gender and included students from the first through fourth years. Their academic fields also varied and included the humanities, social sciences, natural sciences, engineering, education, and law. Thus, the sample did not concentrate in a particular academic field or discipline. Participants reported using conversational AI in varied contexts, including academic assignments, information searching and organisation, writing support, creative activities, and discussions of personal concerns. University A and University B refer to two distinct four-year universities in South Korea. To protect institutional anonymity, we used the labels “A” and “B” rather than the universities’ actual names.

Participants also differed in their frequency of AI use, ranging from fewer than five uses per month to daily use or more than ten uses per week. They used conversational AI for a variety of purposes, including information searching, assignment support, summarising and organising information, writing assistance, creative idea generation, and discussions of personal concerns. These reported purposes indicate that conversational AI was used primarily for academic and informational support, while some participants also used it for emotional or personal conversations.

Defining participants associated with each factor came from diverse demographic backgrounds rather than from a single gender, academic field, or year level. As shown in Table 2, the distribution of academic fields differed somewhat across the four types, but no type was composed exclusively of students from a single field. Type 1 included a relatively large number of engineering and natural science students, whereas Type 2 included students from the social sciences, humanities, law, and natural sciences. Type 3 included humanities students as well as students from engineering, the social sciences, and education, while Type 4 included students from the humanities, natural sciences, social sciences, and engineering. These patterns suggest that academic background may provide useful contextual information for interpreting each type, but the types should not be understood as determined by academic field or discipline. Participants with different levels of AI-use frequency were also represented across the four factors. Because demographic characteristics and usage frequency were not tested as explanatory variables, no causal or statistically generalisable relationship between these characteristics and factor membership is claimed.

Table 2 presents the gender, university, academic field, year level, AI-use frequency, and reported purposes of AI use for the defining participants associated with each factor. These characteristics were used as contextual information to support factor interpretation rather than as variables for statistical generalisation. Further details of the data-processing and factor-analysis procedures are provided in Section 2.3. No Q sorts were classified.

### 2.3. Q-Sorting Data Processing and Analysis

Participants first read all 40 statements and provisionally classified them into three groups: statements with which they agreed, statements with which they disagreed, and statements about which they felt neutral or uncertain. They then placed the statements on a forced-distribution grid ranging from +5 (most agree) to −5 (most disagree), arranging the remaining statements according to their relative agreement or disagreement. We coded the completed Q sorts by assigning scores ranging from −5 to +5 according to the position of each statement. The resulting Q-sort matrix was analysed using KADE version 1.2.1. Principal component analysis was used to extract the initial factors. The number of retained factors was determined by jointly considering eigenvalues, cumulative explained variance, the distribution of Q sorts across factors, the coherence of the factor arrays, and the substantive interpretability of the resulting viewpoints in relation to the research questions. The eigenvalues, explained variance, factor allocation, and factor allocation are reported in Section 3.1.

Varimax rotation was applied to the retained factor solution to improve interpretive clarity and facilitate the construction of factor arrays. The standard error of a factor loading was calculated as 1/√N, where N was the number of Q statements. With 40 statements, the standard error was approximately 0.158, corresponding to significance thresholds of approximately 0.310 at *p* < 0.05 and 0.408 at *p* < 0.01. Cross-loaders were defined as Q sorts that loaded significantly on more than one factor, whereas non-loaders were defined as Q sorts that did not reach the significance threshold on any retained factor. Following Varimax rotation, KADE allocated 22 Q sorts to Factor 1, 14 to Factor 2, 10 to Factor 3, and 10 to Factor 4.

For each retained factor, KADE generated a weighted factor array and statement-specific Z-scores. Distinguishing statements were identified as statements whose Z-scores differed significantly between a given factor and the other factors, whereas consensus statements did not differ significantly across factors. Factor interpretation was based on the highest- and lowest-ranked statements, distinguishing statements, consensus statements, relative-ranking patterns, and the overall configuration of each factor array.

## 3. Results

This section presents the results of the Q-sort analysis of university students’ experiences with conversational AI. Rather than examining the frequency of AI use or reducing participants’ experiences to simple positive or negative evaluations, we focused on how they positioned algorithmic intimacy, social connection, well-being, and autonomy within their subjective meaning structures. Q factor analysis identified four subjective types that differed in how they combined perceived utility, emotional use, relational boundary-setting, concerns about dependence, and the protection of autonomy. The following sections first present the factor extraction results and then interpret the characteristic meaning structure of each type using factor arrays, distinguishing statements, consensus statements, extreme-ranking patterns, and participants’ written post-sort explanations.

### 3.1. Factor Extraction and Type Identification

We conducted Q factor analysis to identify patterns in university students’ subjective perceptions of algorithmic intimacy, social connection, and well-being and autonomy in their experiences with conversational AI. Principal component analysis initially extracted eight unrotated factors. Their eigenvalues were 20.2623, 4.9607, 3.0913, 2.4985, 2.2834, 2.1404, 1.9921, and 1.7502, accounting for 36%, 9%, 6%, 4%, 4%, 4%, 4%, and 3% of the total variance, respectively.

After considering the eigenvalues, cumulative explained variance, the distribution of Q sorts, the coherence of the factor arrays, and the substantive interpretability of the viewpoints, a four-factor solution was retained. The four retained factors jointly explained 55% of the total variance, as shown in Table 3.

The first component explained 36% of the total variance, the second Factor 9%, the third Factor 6%, and the fourth Factor 4%. Although the later Factors also had eigenvalues greater than 1.0, Factors were not retained solely on the basis of the eigenvalue criterion. The final solution was selected by considering statistical adequacy together with the coherence, distinctiveness, and interpretability of the factor arrays. The retained solution also preserved an analytically interpretable autonomy-focused viewpoint represented by Factor 4. Accordingly, the four-factor solution was treated as an interpretable rather than uniquely definitive representation of the data.

Following Varimax rotation, 22 Q sorts defined Factor 1, 14 defined Factor 2, 10 defined Factor 3, and 10 defined Factor 4. No Q sorts were classified as cross-loaders or non-loaders; therefore, all 56 Q sorts were included in the factor interpretation. The four factors were subsequently interpreted as (1) Relationship-Negating Instrumentalists, (2) Selective Emotional Utilizers, (3) Reflective Distancers, and (4) Autonomy Defenders.

These results indicate that university students did not organise their conversational AI experiences along a single continuum of acceptance or rejection. Instead, the four factors represented configurations of subjective meaning that differed in the relative emphasis placed on functional utility, emotional support, relational authenticity, dependence, cognitive autonomy, and the boundaries between AI and human relationships.

### 3.2. Characteristics of Perception Types

#### 3.2.1. Type 1: Relationship-Negating Instrumentalists

Type 1 viewed conversational AI primarily as a practical tool for improving academic efficiency and processing information, while firmly rejecting the possibility that AI could acquire a relational status comparable to that of a human being.

Participants in this type strongly agreed with the statements “AI is a tool that quickly organises the information I need” (Q26, Z = 1.958), “I do not think that conversations with AI can replace human relationships” (Q20, Z = 1.816), “No matter how familiar AI becomes, I do not think it constitutes a real relationship” (Q13, Z = 1.709), “I think that being unable to use AI effectively may put me at an academic disadvantage” (Q35, Z = 1.407), and “I think that intimacy with AI is ultimately an illusion” (Q11, Z = 1.161). They also agreed with “I feel that emotionally relying on AI can be risky” (Q12, Z = 1.117), “I feel uncomfortable when AI provides incorrect information without evidence” (Q31, Z = 1.115), “I view AI as a conversational system rather than as a friend” (Q28, Z = 1.010), and “People who use AI effectively seem more competent” (Q40, Z = 1.007). Taken together, these rankings indicate that Type 1 participants confined AI primarily to functional, informational, and academic roles.

In contrast, participants strongly rejected emotionally and relationally oriented statements, including “I feel that emotional attachment can develop even in a relationship with AI” (Q4, Z = −1.870), “Interacting with AI reduces my loneliness” (Q8, Z = −1.631), “I sometimes feel that AI knows me better than I know myself” (Q3, Z = −1.347), and “When I am having a difficult time, I sometimes want to talk to AI before talking to another person” (Q17, Z = −1.134). This pattern suggests that Type 1 participants did not translate experiences of personalised or responsive AI interaction into relational intimacy. Although they acknowledged AI’s usefulness, they kept it outside the domain of emotional attachment and authentic human relationships. Table 4 presents the most salient statements for Type 1.

The written post-sort explanations provided by participants reinforced this interpretation. P1 stated, “AI is most useful when it quickly organises the information I need.” P12 explained, “When I start an assignment, I first ask AI and then decide on the direction.” P44 similarly stated, “I use AI as a supplementary tool after searching for information.” These explanations show that participants valued AI primarily for accelerating information processing and supporting academic decision-making.

Participants in this type also rejected AI’s relational potential. P15 stated, “AI can only be a machine that provides answers for learning,” and P55 explained, “AI is a tool for handling work efficiently, not an emotional being.” P42 similarly remarked, “AI cannot understand human emotions in the same way that people do,” emphasising that AI did not constitute an authentic relational entity.

An important characteristic of this type is that participants could occasionally experience personalised AI responses as if they were being understood, but they did not extend this experience into relational identification or emotional attachment. For example, P50 explained, “AI responded based on my previous conversations and made suggestions, even though I had not explicitly asked it to analyse me.” This participant reported that AI sometimes appeared to analyse and understand them. However, such experiences were interpreted as evidence of personalised computational functionality rather than genuine relational understanding or intimacy.

In summary, Type 1 participants actively embraced AI’s efficiency and functionality while consistently rejecting emotional attachment and relational authenticity. They viewed AI as a rational and instrumental tool that supported academic work and information processing. For this type, the apparent intimacy generated through personalised responses was interpreted as a product of algorithmic functionality rather than as evidence of a genuine relationship. Accordingly, we named this type ‘Relationship-Negating Instrumentalists.’

#### 3.2.2. Type 2: Selective Emotional Utilizers

Type 2 includes students who do not view conversational AI as a substitute for human relationships but who use it as a practical resource for emotional regulation and self-disclosure. Although these students do not regard AI as a fully relational entity, they experience it as an auxiliary conversational partner that helps them organise their emotions, express difficult thoughts, and obtain temporary emotional relief. Therefore, they use AI selectively for emotional self-disclosure and affective regulation rather than as a replacement for human relationships.

The Q-sort results reflected this perception structure. Participants in this type strongly agreed with statements such as “I can tell AI things that are difficult to say to other people” (Q7, Z = 2.261), “I feel uncomfortable when AI provides incorrect information without evidence” (Q31, Z = 1.731), “AI is a tool that quickly organises the information I need” (Q26, Z = 1.624), “AI helps me organise my emotions” (Q9, Z = 1.557), “I think that conversations with AI can sometimes help improve my relationships with other people” (Q21, Z = 1.413), and “Conversations with AI comfort me” (Q15, Z = 1.007). These responses indicate that Type 2 participants simultaneously valued AI’s emotional usefulness and its practical functionality while retaining a critical awareness of its informational limitations.

By contrast, this type showed low agreement with statements such as “When I use AI, I sometimes find interacting with people bothersome” (Q34, Z = −1.958), “I feel empty when I am unable to converse with AI” (Q5, Z = −1.543), “I feel that conversing with AI is becoming habitual for me” (Q18, Z = −1.538), “Using AI sometimes makes me feel more emotionally anxious” (Q10, Z = −1.263), and “When I follow AI’s advice, I sometimes feel that my own range of choices becomes narrower” (Q39, Z = −1.252). These responses suggest that although Type 2 recognised AI’s emotional value, participants did not strongly associate their AI use with emotional emptiness, habitual dependence, interpersonal avoidance, or a loss of autonomy. Table 5 presents the statements with which Type 2 most strongly agreed and disagreed.

The written post-sort explanations further supported this interpretation. P23 stated, “It is easier to talk about things that are not highly confidential but still feel burdensome to discuss with other people.” Similarly, P24 explained, “I do not think I can say everything to my friends, but with AI, I can talk without worrying because it does not judge me.” These comments indicate that participants experienced AI as a non-judgemental and readily accessible channel for emotional expression.

At the same time, participants in this type remained sensitive to the possibility of AI-generated errors. P45 stated, “When AI provided incorrect information without verification, I searched again to check it.” This response shows that participants used AI cautiously and retained critical judgement when interacting with it. Thus, for Type 2, AI functioned as an auxiliary space for organising emotions and facilitating self-expression. In this respect, Type 2 accepted algorithmic intimacy selectively and within defined boundaries, interpreting it primarily as a means of self-disclosure and emotional regulation rather than as evidence of a fully reciprocal relationship.

In summary, Type 2 participants experienced algorithmic intimacy but maintained clear boundaries around that experience. They actively embraced the emotional benefits of AI while showing relatively low identification with dependence, interpersonal withdrawal, emotional anxiety, or diminished autonomy. Rather than treating AI as equivalent to a human relationship, they used it as a supplementary and non-judgemental channel for emotional expression. Accordingly, we labelled this type ‘Selective Emotional Utilizers’.

#### 3.2.3. Type 3: Reflective Distancers

Type 3 includes students who acknowledge the functional usefulness of conversational AI while remaining cautious about the possibility that its convenience and responsiveness could be interpreted as emotional intimacy or could foster cognitive dependence. These students actively use AI while simultaneously reflecting on its non-human nature, its relational limitations, and its potential influence on independent thinking.

The Q-sort results reflected this perception structure. Participants in this type strongly agreed with statements such as “I do not think that conversations with AI can replace human relationships” (Q20, Z = 1.895), “I view AI as a conversational system rather than as a friend” (Q28, Z = 1.666), and “AI is a tool that quickly organises the information I need” (Q26, Z = 1.439). They also agreed with “I worry that AI may narrow my thinking by providing answers that align mainly with my existing preferences” (Q32, Z = 1.401), “Conversations with AI sometimes feel helpful and emotionally empty at the same time” (Q27, Z = 1.297), and “I feel that emotionally relying on AI can be risky” (Q12, Z = 1.284). These responses indicate that Type 3 participants simultaneously recognised AI’s practical benefits, relational limitations, and potential cognitive risks.

By contrast, participants strongly disagreed with statements such as “I feel empty when I am unable to converse with AI” (Q5, Z = −2.143), “I sometimes feel that AI knows me better than I know myself” (Q3, Z = −1.804), “I sometimes feel disappointed when AI’s responses do not meet my expectations” (Q38, Z = −1.485), and “When I use AI, I sometimes find interacting with people bothersome” (Q34, Z = −1.269). These responses suggest that Type 3 participants did not experience the absence of AI as an emotional loss, did not attribute superior self-understanding to AI, and did not report strong emotional expectations of its responses. Table 6 summarises the statements with which Type 3 most strongly agreed and disagreed.

The written post-sort explanations provided a more concrete understanding of this perception structure. P5 stated, “Even after searching, the first place I turn to is not ChatGPT but Naver,” indicating that this participant positioned AI as a secondary rather than primary information source. P25 explained, “AI is only a familiar tool; it cannot become a real relationship in the same way as a relationship with another person.” These explanations show that Type 3 participants valued AI’s usefulness while maintaining a clear distinction between technical interaction and human relationships.

Compared with Type 1, Type 3 displayed a more explicitly reflective and ambivalent stance toward AI use. Type 1 primarily confined AI to an instrumental role and rejected its relational significance. Type 3 similarly maintained a boundary between AI and human relationships but paid greater attention to the psychological and cognitive effects that could arise through repeated interaction. In particular, participants recognised that AI could be useful while also feeling emotionally empty during the interaction itself (Q27), yet they did not report feeling empty when AI was unavailable (Q5). This distinction indicates situational ambivalence about the quality of AI interaction rather than emotional dependence on its continued availability.

Type 3 also expressed concern that personalised answers could reinforce existing preferences and narrow independent thinking (Q32), while recognising the risks of emotional reliance (Q12) and becoming concerned when dependence was noticed (Q22). Their distancing therefore did not represent rejection or avoidance of AI use. Rather, it reflected deliberate monitoring of how AI might influence their emotional responses, writing habits, judgments, and patterns of thought.

In summary, Type 3 participants used AI as a valuable practical tool while critically examining the possibility that personalised interaction could be misinterpreted as intimacy or could constrain independent thinking. They neither rejected AI use nor accepted algorithmic intimacy uncritically. Instead, they experienced conversational AI through a combination of utility, situational emotional ambivalence, and reflective boundary-setting. Accordingly, we named this type ‘Reflective Distancers.’

#### 3.2.4. Type 4: Autonomy Defenders

Type 4 includes students who acknowledge the functional efficiency and practicality of conversational AI but place stronger emphasis on the risk that AI use may weaken independent thinking, judgement, decision-making, and self-regulation. Although these students use AI as a practical tool, they prioritise setting boundaries around its use and protecting autonomy over developing emotional dependence or relational attachment. Thus, they interpret conversational AI experiences primarily through the lens of self-determination and cognitive autonomy rather than emotional intimacy.

The Q-sort results reflected this perception structure. Participants in this type strongly agreed with statements such as “I do not think that conversations with AI can replace human relationships” (Q20, Z = 1.931), “I worry that using AI too much may weaken my ability to think for myself” (Q33, Z = 1.876), “AI is a tool that quickly organises the information I need” (Q26, Z = 1.636), “No matter how familiar AI becomes, I do not think it constitutes a real relationship” (Q13, Z = 1.550), “I feel that emotionally relying on AI can be risky” (Q12, Z = 1.339), and “When I notice myself relying on AI, I become concerned about that reliance” (Q22, Z = 1.016). These responses indicate that Type 4 participants recognised AI’s efficiency while maintaining heightened vigilance regarding its potential effects on independent thought and self-directed decision-making.

By contrast, Type 4 strongly disagreed with statements such as “I feel more comfortable talking with AI than talking with people” (Q19, Z = −1.691), “When I am having a difficult time, I sometimes want to talk to AI before talking to another person” (Q17, Z = −1.823), “When talking with AI, there are times when I lose track of time” (Q16, Z = −1.355), “I feel empty when I am unable to converse with AI” (Q5, Z = −1.354), and “I feel that conversing with AI is becoming habitual for me” (Q18, Z = −1.201). This pattern suggests that Type 4 participants did not strongly identify with habitual use, emotional substitution, interpersonal preference for AI, or absorption in AI interaction. Instead, they sought to maintain control over when and how AI influenced their thinking and behaviour. Table 7 presents the salient statements for Type 4.

The written post-sort explanations further clarified this autonomy-oriented perception structure. P39 stated, “There are times when I use AI out of laziness, even for assignments that I could complete on my own if I invested enough time,” but also explained, “The more I use AI, the more I worry that I may rely on it instead of thinking for myself.” Similarly, P54 stated, “The more I use AI, the fewer opportunities I may have to think independently.” These explanations reflect concern that AI’s convenience could gradually reduce opportunities for autonomous thought and problem-solving.

Some participants also acknowledged that AI could provide limited emotional comfort in particular situations. For example, P33 stated, “When I am alone, talking with AI can sometimes help relieve feelings of emptiness.” However, this individual explanation should be interpreted in conjunction with the factor-level pattern. Type 4 as a whole disagreed with both feeling empty when AI was unavailable (Q5) and experiencing AI conversations as simultaneously helpful and emotionally empty (Q27). Therefore, occasional situational comfort did not amount to a shared pattern of emotional dependence or relational identification within this type.

Participants instead maintained a firm distinction between AI interaction and reciprocal human relationships. P36 explained, “Relationships require shared experiences and reciprocity, whereas AI is only a system that operates according to a programme.” P40 emphasised, “Conversations with AI are useful, but communication with humans is fundamentally different.” P43 also referred to the rapid development of AI and the uncertainty surrounding legal and normative standards, stating, “I do not know how far its use should be allowed.” These explanations demonstrate that Type 4 participants extended their concern beyond personal dependence to include broader questions of technological boundaries, responsibility, and appropriate regulation.

Compared with Type 3, Type 4 expressed a more direct and protective orientation toward autonomy. Type 3 reflected on the ambiguity, emotional emptiness, and cognitive risks of AI interaction, whereas Type 4 placed stronger emphasis on actively preventing AI from weakening independent thinking and self-determination. Their position was therefore not simply one of reflective distance but of deliberate autonomy protection.

In summary, Type 4 participants acknowledged AI’s efficiency and practicality while remaining especially cautious about the possibility that AI use could weaken cognitive capacity, independent judgement, or self-directed choice. Although some participants recognised limited situational comfort, the factor-level pattern did not indicate emotional reliance or relational attachment. For these students, algorithmic intimacy represented a context in which the boundaries of technological influence had to be actively managed in order to protect personal agency and cognitive autonomy. Accordingly, we labelled this type ‘Autonomy Defenders’.

#### 3.2.5. Comparative Analysis of Perception Types

All four types shared the belief that conversational AI cannot fully replace human relationships. This shared boundary was most clearly reflected in the consistently high ranking of Q20, “I do not think that conversations with AI can replace human relationships,” across the four factor arrays. However, they differed in how they interpreted AI’s functional usefulness, accepted algorithmic intimacy, maintained relational boundaries, and evaluated the influence of AI use on autonomy and self-determination.

To present the differences among the four types more clearly, the characteristics of each type are comparatively summarised in Table 8.

The comparative pattern becomes clearer when the consensus, distinguishing, and extreme-ranking statements are considered together. The comparative pattern becomes clearer when shared ranking tendencies, distinguishing statements, and extreme-ranking statements are considered together. Across the four factor arrays, Q20 was consistently ranked positively, indicating a shared boundary between conversational AI and human relationships. However, their distinguishing statements revealed different grounds for maintaining this boundary. Type 1 was distinguished by its strong endorsement of academic utility (Q35) and its rejection of emotional attachment and loneliness reduction (Q4, Q8, and Q17). Type 2 was distinguished by its willingness to disclose difficult thoughts to AI (Q7), its perception that AI could support human relationships (Q21), and its rejection of interpersonal withdrawal (Q34). Type 3 was distinguished by concern that personalised answers could narrow thinking (Q32), by the simultaneous experience of usefulness and emotional emptiness (Q27), and by its rejection of absence-related emptiness (Q5). Type 4 was distinguished by its strong concern that excessive AI use could weaken independent thinking (Q33) and by its rejection of turning to AI before other people in difficult situations (Q17).

The extreme-ranking patterns further differentiated the four types. Type 1 placed functional utility and the irreplaceability of human relationships at the positive extreme, while placing emotional attachment and loneliness reduction at the negative extreme. Type 2 placed self-disclosure and concern about inaccurate information at the positive extreme, while placing interpersonal avoidance and absence-related emptiness at the negative extreme. Type 3 placed the irreplaceability of human relationships and the classification of AI as a conversational system at the positive extreme, while strongly rejecting absence-related emptiness and the belief that AI knows the self better than the user does. Type 4 placed the irreplaceability of human relationships and concern about weakened independent thinking at the positive extreme, while strongly rejecting a preference for AI over people and the tendency to turn to AI first during difficult experiences.

Taken together, the four types differed in the relative importance they assigned to functional utility, emotional self-disclosure, relational authenticity, cognitive risk, and the protection of autonomy.

## 4. Discussion

This study found that university students do not simply accept or reject conversational AI. Instead, they construct distinct subjective meaning structures based on the extent to which they accept algorithmic intimacy and establish relational boundaries with AI. Although all four types agreed that AI cannot completely replace human relationships, they differed markedly in how they defined psychological boundaries and regulated their relationships with AI. These findings suggest that human–AI relationships in the era of generative AI can be understood as processes of subjective meaning construction through which users negotiate intimacy, social connection, well-being, and autonomy.

The four types identified in this study do not represent a continuum of approval or disapproval towards AI. Rather, they reflect distinct meaning structures shaped by the extent to which users permit intimacy and protect autonomy. Relationship-Negating Instrumentalists (Type 1) embraced AI’s efficiency and functionality while firmly denying it any relational status. Selective Emotional Utilizers (Type 2) maintained the premise that AI cannot replace human relationships but selectively used it as a non-judgemental and accessible resource for emotional regulation. Reflective Distancers (Type 3) recognised AI’s usefulness while remaining aware of the emotional ambiguity and potential cognitive dependence associated with AI interaction. Autonomy Defenders (Type 4) valued AI’s practical utility but strongly cautioned against the possibility of weakened thinking, reduced self-regulation, and diminished self-determination. These findings indicate that the central issue is not whether users develop algorithmic intimacy, but how they interpret that intimacy, position it in relation to human relationships, and define its boundaries.

These findings also provide important insights into the relationship between AI and social connection. Type 2 participants viewed AI as an auxiliary space in which they could express emotions that they found difficult to share with others, suggesting that AI-mediated interaction may support emotional self-disclosure without necessarily substituting for human relationships. By contrast, Types 1, 3, and 4 maintained a clear distinction between AI interaction and human interaction. Conversational AI was therefore interpreted variously as a supplementary resource, a non-relational tool, a context for reflective distancing, or a potential risk to autonomy and human relationships. The relationship between AI and social connection should thus be understood through the relational boundaries users establish, rather than solely through replacement or supplementation.

Reeves and Nass’s (1996) media equation theory argues that humans tend to respond to technologies as if they were social actors. This theory helps explain why conversational AI can be experienced as responsive, socially present, and emotionally meaningful, even when users know that AI does not possess genuine emotions or intentions. The present findings, however, show that such social responses are interpreted differently across the four types. Type 2 most clearly reflects the media equation logic: these participants accepted AI’s responsive and non-judgmental interaction as an emotional resource for self-disclosure and temporary support. Type 3, by contrast, recognised AI’s social responsiveness but cognitively deconstructed it, interpreting the resulting intimacy as useful yet incomplete or empty. Type 1 limited AI’s apparent sociality to functional usefulness and denied its relational status, whereas Type 4 interpreted the same responsiveness through concerns about autonomy, dependence, and the protection of independent thinking. Thus, the study extends media equation theory by showing that socially responsive technology does not produce a uniform relational outcome. Social responses to AI may be accepted, selectively utilised, critically questioned, or actively constrained according to users’ subjective meaning structures and relational boundaries. Algorithmic intimacy should therefore be understood not as a relationship that automatically deepens through technological responsiveness, but as an experience that users interpret, permit, and regulate.

These findings also connect to the discussion of parasocial relationships. Horton and Wohl (1956) and Liebers and Schramm (2019) explain how individuals develop one-sided intimacy with media figures or virtual agents. The present study shows that participants interpreted such intimacy in different ways. Type 2 participants tended to use parasocial intimacy as a source of emotional support, whereas Types 3 and 4 treated AI-mediated intimacy as an opportunity for reflection and boundary setting. Type 1 participants rejected the possibility of forming a relationship with AI altogether. These patterns suggest that the central issue in relationships with conversational AI is how users perceive AI-mediated intimacy and the extent to which they regard it as emotionally meaningful, relationally acceptable, or in need of limitation. Furthermore, the findings suggest that AI-mediated intimacy does not necessarily function as a substitute for human relationships. Instead, users may interpret it as a supplementary source of emotional support or as a limited form of interaction that remains distinct from human relationships, depending on how they establish relational boundaries.

Attachment theory (Mikulincer & Shaver, 2016) also provides a useful lens for interpreting these findings. Some students, particularly those in Type 2, experienced AI as a temporary, secure-base-like source of immediate and non-judgemental support. This experience aligns with the self-disclosure facilitation effects suggested by Lucas et al. (2014) and Ho et al. (2018). However, Types 3 and 4 recognised that this secure-base function could turn into dependence and therefore maintained a critical distance from AI. As a result, AI did not function uniformly as an object of attachment but differed depending on the user’s boundary awareness and self-regulation strategies. In this respect, the findings are consistent with arguments that conversational AI may be experienced as a source of emotional support, while also showing that users do not uniformly interpret such support as attachment or dependence.

Recent studies have reported that AI companions may promote well-being (Nakagomi et al., 2026; Ueda et al., 2026). Recent studies have reported that AI companions may promote well-being (Nakagomi et al., 2026; Ueda et al., 2026). In the present study, some participants, particularly those in Type 2, perceived AI as helping them regulate emotions or reduce loneliness. However, well-being-related interpretations differed across types. Type 2 participants used AI as a resource that complemented social connections. Type 3 participants experienced AI ambivalently, perceiving both usefulness and emotional emptiness. Type 4 participants expressed concern that excessive AI use could undermine well-being and autonomy, whereas Type 1 participants rarely regarded AI as a meaningful source of well-being. Thus, the relationship between AI and well-being was not linear. Rather than demonstrating a direct effect on well-being, the findings show that students interpreted AI in relation to emotional regulation, dependence, and autonomy in different ways.

Recent literature on LLM-based conversational agents in mental healthcare helps clarify these type-specific differences. Shankar et al. (2026) note that LLM-based conversational agents may improve access to mental health support through availability, scalability, and reduced barriers to disclosure, but they also identify important implementation concerns, including safety deficiencies, crisis detection, privacy, and the need for appropriate safeguards. Although the present study did not examine the clinical use of conversational AI, this tension is relevant to the present findings. Type 2 participants’ use of AI for emotional self-disclosure and temporary support corresponds to facilitators of LLM-mediated emotional support identified in this literature, particularly accessibility, immediacy, and reduced fear of interpersonal judgment. By contrast, Type 3 participants’ sense of usefulness mixed with emptiness, and Type 4 participants’ concerns about dependence and autonomy, correspond to concerns associated with such systems, including uncertainty about authenticity, over-reliance, and the need for boundary-setting. Thus, the present study contributes to the emerging literature on LLM-mediated mental well-being by showing that the same affordances that make conversational AI emotionally accessible can also generate ambivalence, dependence concerns, and demands for relational and ethical boundaries.

By revealing these patterns, this study moves beyond research that approaches algorithmic intimacy, social connection, well-being, and autonomy as separate variables. It shows that these dimensions form interconnected interpretive structures. The key issue in human–AI relationships is not whether AI use increases or decreases well-being, but how users understand their interactions with AI and assign relational meaning to them. In this respect, the study shifts attention from whether AI-mediated intimacy exists to how users negotiate its boundaries.

The emergence of four distinct perception structures among students who use the same technology further suggests that human–AI relationships do not arise solely from the technology’s characteristics. The Korean university context may help explain why academic utility, emotional regulation, relational boundary-setting, and autonomy concerns were strongly intertwined in the four types identified in this study. In South Korea, the rapid adoption of digital technologies and generative AI means that students encounter conversational AI not as a marginal or experimental tool but as an increasingly ordinary resource for academic work, career preparation, information processing, and self-expression. At the same time, intense academic competition, employment uncertainty, evaluation pressure, relationship fatigue, and emotional isolation may make AI attractive as a low-risk space for self-disclosure and emotional regulation (Organisation for Economic Co-Operation and Development [OECD], 2025; Jun, 2024; Y. S. Yang, 2026; H. Lee et al., 2026). The same context may also heighten concerns about dependence, authorship, ethical use, and the weakening of independent thinking. Because Korean universities are still developing stable norms and assessment standards for generative AI use, students negotiate AI use in an environment where efficiency and anxiety coexist (Korean Council for University Education, 2025). The four types should therefore be interpreted as contextually situated rather than culturally determined or universally fixed categories. The Korean context does not directly produce these types; rather, it provides conditions in which academic utility, emotional self-management, relational boundaries, and autonomy become especially salient and interconnected. In addition, the findings suggest that AI-mediated intimacy involves more than emotional or technological processes. Users also connect it to questions of agency and autonomy. By highlighting this connection, the study expands the theoretical scope of human–AI relationship research.

The findings also carry important implications for higher education. Universities should not treat generative AI solely as an academic support tool or as a source of academic misconduct. While students use AI to organise information, they also use it to regulate emotions, disclose personal concerns, process relational stress, and enhance self-understanding. Therefore, AI literacy education should go beyond technical skills and encourage students to manage relational distance from AI, recognise signs of dependence, reflect on differences between AI and human relationships, and exercise ethical judgement.

Universities should tailor AI literacy education to students’ different patterns of AI use. For students in Type 1, who perceive AI primarily as a functional tool, educators should emphasise critical-use skills such as identifying information errors, recognising bias, and applying ethical standards. For students in Type 2, who use AI as an emotional resource, educators should provide concrete guidance on emotional self-disclosure, privacy protection, and the appropriate use of AI for emotional support. Such guidance should encourage students to distinguish between low-risk emotional expression and the disclosure of highly sensitive personal information, to avoid entering identifiable data or intimate details into commercial AI systems, and to understand how conversation data may be stored, processed, or reused. Educators should also help students recognise when AI-based emotional support should be supplemented by human support, such as counselling services, peer relationships, or professional help. For students in Type 3, educators should promote metacognitive strategies that encourage reflection on the emotional limitations of AI interaction, the authenticity of AI-mediated intimacy, and the possibility of cognitive dependence. For students in Type 4, educators should strengthen critical-use skills that help students maintain independent thinking and self-determination while interacting with AI. For AI developers, these findings suggest the need for privacy-by-design approaches, transparent data-use notices, user-friendly controls for deleting or limiting conversation histories, and safeguards that remind users not to disclose sensitive personal information during emotionally oriented interactions. Emotionally oriented systems should also include clear pathways directing users towards appropriate human or professional support when serious distress or crisis-related content is detected.

Taken together, the findings show that algorithmic intimacy is not a uniform psychological outcome but a negotiated experience interpreted through users’ emotional needs, social connections, relational boundaries, critical awareness, and efforts to preserve autonomy.

## 5. Conclusions

This study explored university students’ experiences with conversational AI from the perspectives of algorithmic intimacy, social connection, well-being, and autonomy and identified their subjective meaning structures using Q methodology. The analysis identified four types: Relationship-Negating Instrumentalists, Selective Emotional Utilizers, Reflective Distancers, and Autonomy Defenders. Although all four types maintained a distinction between AI interaction and human relationships, they differed in how they combined AI’s functional utility, emotional value, perceptions of relational authenticity, feelings of emptiness, concerns about dependence, and the protection of autonomy.

The findings indicate that university students do not simply accept or reject intimacy with AI. Rather, they negotiate how much relational meaning to attribute to AI and where to establish emotional, relational, and cognitive boundaries. Conversational AI may function as an efficient tool, a supplementary channel for emotional self-disclosure, an interaction that is useful yet emotionally limited, or a potential threat to independent thinking. Algorithmic intimacy should therefore be understood as a negotiated and regulated experience rather than as a uniform psychological outcome.

The findings position conversational AI not as a direct determinant of young adults’ identity or autonomy, but as a mediating environment in which users evaluate whether their emotions, judgements, and choices remain under their own control. This interpretation highlights the interdependence of intimacy, social connection, well-being, and autonomy in human–AI interaction.

These results have implications for higher education. AI literacy education should extend beyond technical proficiency, prompt construction, information verification, and academic integrity. Universities should also help students distinguish AI-mediated interaction from reciprocal human relationships, manage emotional self-disclosure, protect privacy, recognise potential dependence, maintain critical judgement, and preserve cognitive autonomy. Ultimately, understanding conversational AI requires attention not only to whether users experience intimacy with AI, but also to how they interpret, limit, and regulate that intimacy in everyday life.

## 6. Limitations and Future Research

This study has the following limitations.

First, this exploratory Q-methodological study involved university students from two South Korean universities. Therefore, the four types should be understood as contextually situated subjective viewpoints rather than as statistically generalisable or universally applicable categories. The Korean university context may have made academic efficiency, emotional self-management, and autonomy concerns particularly salient, but this study did not directly compare Korean students with participants from other cultural, age, or occupational groups. Future research should examine whether similar or different subjective types emerge across broader populations and cultural contexts.

Second, the study relied on cross-sectional Q-sort data and participants’ written post-sort explanations. It did not directly examine actual AI-use behaviour, platform-use histories, or changes in well-being and autonomy over time. The identified types should therefore be interpreted as subjective positions rather than as stable behavioural traits or evidence of causal psychological effects. Longitudinal and mixed-methods studies combining repeated Q sorts, AI-use logs, experience-sampling data, and follow-up interviews could examine how these perceptions change with accumulated AI experience.

Third, the factor structure should be interpreted cautiously. Factor 1 explained a considerably larger proportion of variance than the other factors, and some inter-factor relationships were relatively strong. The four-factor solution should therefore be regarded as an interpretable representation of overlapping but substantively distinguishable configurations of meaning, rather than as a uniquely definitive or mutually exclusive classification. Future studies should examine the robustness of the factor structure using alternative samples, factor-retention criteria, factor solutions, and rotation methods.

Fourth, this study did not systematically account for differences among conversational AI platforms. Features such as conversational memory, personalised responses, anthropomorphic language, privacy settings, and safety mechanisms may influence users’ experiences of intimacy, dependence, and autonomy. Future research should compare specific platforms and interaction designs, as well as other AI forms such as social robots, virtual companions, and embodied agents.

Taken together, future research should examine the contextual transferability, temporal stability, and technological sensitivity of the subjective meaning structures identified in this study.

## Figures and Tables

**Table 1:** Q sample.

No.	Statement	Primary Analytic Dimension	Valence
Q1	AI makes me feel that in understands me well.	Algorithmic intimacy	Positive
Q2	My relationship with AI is becoming increasingly familiar.	Algorithmic intimacy	Positive
Q3	I sometimes feel that AI knows me better than I know myself.	Algorithmic intimacy	Negative/ Concern oriented
Q4	I feel that emotional attachment can develop even in a relationship with AI.	Algorithmic intimacy	Positive
Q5	I feel empty when I am unable to converse with AI.	Well-being and emotional regulation	Negative/ Concern oriented
Q6	Interaction with AI can distance me from my relationships with other people.	Social connection	Negative/ Concern oriented
Q7	I can tell AI things that are difficult to say to other people.	Social connection	Positive
Q8	Interacting with AI reduces my loneliness.	Social connection	Positive
Q9	AI helps me organise my emotions.	Well-being and emotional regulation	Positive
Q10	Using AI sometimes makes me feel more emotionally anxious.	Well-being and emotional regulation	Negative/ Concern oriented
Q11	I think intimacy with AI is ultimately an illusion.	Algorithmic intimacy	Negative/ Concern oriented
Q12	I feel that emotionally relying on AI can be risky.	Well-being and emotional regulation	Negative/ Concern oriented
Q13	No matter how familiar AI becomes, I do not think it constitutes a real relationship.	Algorthemic intimacy	Ambivalent/ Boundary setting
Q14	AI feels like a non-judgemental conversational partner.	Algorithmic intimacy	Positive
Q15	Conversations with AI comfort me.	Well-being and emotional regulation	Positive
Q16	When talking with AI, there are times when I lose track of time.	Algorithmic intimacy	Negative/ Concern oriented
Q17	When I am having a difficult time, I sometimes want to talk to AI before talking to another person.	Social connection	Positive
Q18	I feel that conversing with AI is becoming habitual for me.	Algorithmic intimacy	Negative/ Concern oriented
Q19	I feel more comfortable talking with AI than talking with people.	Social connection	Negative/ Concern oriented
Q20	I do not think that conversations with AI can replace human relationships.	Social connection	Ambivalent/ Boundary setting
Q21	I think that conversations with AI can sometimes help improve my relationships with other people.	Social connection	Positive
Q22	When I notice myself relying on AI, I become concerned about that reliance.	Well-being and emotional regulation	Negative/ Concern oriented
Q23	I am still unsure how to conceptualise AI.	Well-being and emotional regulation	Ambivalent/ Boundary-setting
Q24	I have not yet fully made sense of what my relationship with AI means to me.	Algorithmic intimacy	Ambivalent/ Boundary-setting
Q25	When doing assignments, AI is the first thing that comes to mind.	Boundaries of autonomy	Positive
Q26	AI is a tool that quickly organises the information I need.	Boundaries of autonomy	Positive
Q27	Conversations with AI sometimes feel helpful and emotionally empty at the same time.	Well-being and emotional regulation	Ambivalent/ Boundary-setting
Q28	I view AI as a conversational system rather than as a friend.	Algorithmic intimacy	Ambivalent/ Boundary-setting
Q29	Using AI can affect my emotional state.	Well-being and emotional regulation	Ambivalent/ Boundary-setting
Q30	Using AI has made me less careful when writing.	Boundaries of autonomy	Negative/ Concern oriented
Q31	I feel uncomfortable when AI provides incorrect information without evidence.	Boundaries of autonomy	Negative/ Concern oriented
Q32	I worry that AI may narrow my thinking by providing answers that align mainly with my existing preferences.	Boundaries of autonomy	Negative/ Concern oriented
Q33	I worry that using AI too much may weaken my ability to think for myself.	Boundaries of autonomy	Negative/ Concern oriented
Q34	When I use AI, I sometimes find interacting with people bothersome.	Social connection	Negative/ Concern oriented
Q35	I think that being unable to use AI effectively may put me at an academic disadvantage.	Boundaries of autonomy	Positive
Q36	I worry that using AI for assignments may raise ethical concerns.	Boundaries of autonomy	Negative/ Concern oriented
Q37	AI sometimes seems to respond as though it intends to care for me.	Algorithemic intimacy	Positive
Q38	I sometimes feel disappointed when AI’s responses do not meet my expectations.	Well-being and emotional regulation	Negative/ Concern oriented
Q39	When I follow AI’s advice, I sometimes feel that my own range of choices becomes narrower.	Boundaries of autonomy	Negative/ Concern oriented
Q40	People who use AI effectively seem more competent.	Boundaries of autonomy	Positive

**Table 2:** Characteristics of Participants Defining Each Factor.

Factor	No.	Factor Weight	Gender	University	Academic Field	Year Level	Frequency of AI Use	Purpose of Use
1	1	10.0000	Female	B	Education	1	1–3 times per week	Information searching, organising information
	15	7.64166	Male	A	Social Sciences	2	1–3 times per week	Information searching
	50	7.39405	Male	B	Engineering	1	1–3 times per week	Assignments
	55	7.09876	Male	B	Engineering	1	1–3 times per week	Information searching, organising information
	12	6.6238	Male	A	Social Sciences	1	4–6 times per week	Assignments, information searching
	44	6.55463	Male	B	Engineering	3	4–6 times per week	Assignments, solving exam questions, mathematical formulas
	49	6.47103	Male	B	Engineering	4	1–3 times per week	Assignments, papers, information searching
	18	6.27371	Female	A	Humanities	2	4–6 times per week	Information searching, organising information
	42	6.23192	Male	A	Humanities	2	7–10 times per week	Assignments
	29	6.0569	Male	B	Engineering	3	4–6 times per week	Information searching, organising information
	34	5.21466	Female	B	Natural Sciences	1	1–3 times per week	Information searching
	22	4.57379	Male	B	Engineering	1	4–6 times per week	Information searching
	56	4.22555	Female	B	Engineering	4	4–6 times per week	Information searching
	21	4.15961	Male	A	Natural Sciences	1	Daily	Mathematical formulas, image editing
	8	3.87874	Female	B	Natural Sciences	1	Fewer than 5 times per month	Health information
	51	3.85398	Male	B	Engineering	1	7–10 times per week	Learning
	47	3.78712	Male	B	Engineering	3	7–10 times per week	Assignments
	3	3.36379	Male	A	Education	3	Daily	Self-study
	14	3.29665	Female	A	Engineering	1	4–6 times per week	Information searching
	48	3.12977	Male	B	Engineering	4	1–3 times per week	Information searching, organising information
	7	2.66166	Female	A	Social Sciences	1	4–6 times per week	Scheduling, organising information, creative idea generation
	30	2.62938	Female	A	Social Sciences	1	1–3 times per week	Self-study
2	20	13.412	Female	A	Social Sciences	2	Daily	Assignments, email writing, discussing personal concerns
	23	8.9962	Female	A	Social Sciences	2	1–3 times per week	Fortune-telling, discussing personal concerns
	45	8.95282	Female	B	Natural Sciences	1	4–6 times per week	Psychological support, career counselling, discussing personal concerns
	17	6.45037	Female	B	Social Sciences	3	Daily	Certification exam preparation
	24	5.23569	Female	B	Natural Sciences	1	1–3 times per week	Writing, discussing personal concerns
	26	4.65785	Female	A	Humanities	2	4–6 times per week	Information searching
	35	4.63344	Male	A	Humanities	2	4–6 times per week	Discussing personal concerns, conversation
	9	4.42517	Female	A	Social Sciences	1	More than 10 times per week	Question resolution, information verification
	10	4.20248	Male	A	Humanities	1	1–3 times per week	Cooking, cleaning, household inquiries, writing assistance
	32	3.84754	Female	A	Law	2	Fewer than 5 times per month	Visualization, reference searching
	11	3.06367	Female	A	Humanities	2	7–10 times per week	Assignments, saju-based fortune-telling
	31	3.03722	Female	A	Law	2	4–6 times per week	Information searching
	28	2.89449	Female	B	Social Sciences	3	4–6 times per week	Assignments, saju-based fortune-telling
	4	2.35803	Female	A	Humanities	3	1–3 times per week	Information searching, objective decision-making
3	16	9.03555	Female	A	Humanities	1	4–6 times per week	Information searching
	5	7.36555	Male	B	Engineering	3	4–6 times per week	Information searching, organising information
	13	5.46386	Female	A	Humanities	1	4–6 times per week	Information searching
	46	5.03658	Female	A	Social Sciences	1	1–3 times per week	Writing feedback
	41	5.00767	Female	A	Education	1	1–3 times per week	Information searching
	25	4.40379	Male	A	Humanities	1	1–3 times per week	Information summarisation, sentence revision
	27	4.28249	Female	A	Humanities	3	1–3 times per week	Assignments, studying
	53	3.39022	Male	B	Social Sciences	3	4–6 times per week	Assignments, discussing personal concerns
	2	3.16328	Male	A	Humanities	2	4–6 times per week	Creative writing
	19	2.46404	Male	B	Engineering	3	4–6 times per week	Assignments, mathematical formulas
4	39	8.45402	Male	A	Humanities	2	4–6 times per week	Assignments
	37	5.88449	Male	A	Natural Sciences	1	4–6 times per week	Information searching
	33	5.74948	Male	A	Humanities	1	4–6 times per week	Information searching
	40	5.00225	Male	B	Natural Sciences	2	1–3 times per week	Information searching, discussing personal concerns
	54	4.83368	Female	B	Humanities	1	4–6 times per week	Information searching, fortune-telling
	43	4.34035	Female	A	Social Sciences	1	1–3 times per week	Information searching
	36	3.51224	Female	A	Humanities	2	7–10 times per week	Information searching
	38	3.32648	Male	B	Engineering	3	4–6 times per week	Information searching
	52	2.94529	Female	B	Social Sciences	1	4–6 times per week	Text summarisation, information searching
	6	2.81407	Male	A	Engineering	2	Fewer than 5 times per month	Information searching, assignments

**Table 3:** Eigenvalues and Explained Variance of the Unrotated Factors.

	Factor 1	Factor 2	Factor 3	Factor 4	Factor 5	Factor 6	Factor 7	Factor 8
Eigenvalues	20.2623	4.9607	3.0913	2.4985	2.2834	2.1404	1.9921	1.7502
% Explained Variance	36	9	6	4	4	4	4	3
Cumulative % Explained Variance	36	45	51	55	59	63	67	70

**Table 4:** Salient statements for Type 1 with Z-Scores ≥ 1.00 or ≤−1.00.

No.	Statement	Z-Score	Q Value
26	AI is a tool that quickly organises the information I need.	1.958	5
20	I do not think that conversations with AI can replace human relationships.	1.816	5
13	No matter how familiar AI becomes, I do not think it constitutes a real relationship.	1.709	4
35 *	I think that being unable to use AI effectively may put me at an academic disadvantage.	1.407	4
11 *	I think intimacy with AI is ultimately an illusion.	1.161	4
12	I feel that emotionally relying on AI can be risky.	1.117	3
31	I feel uncomfortable when AI provides incorrect information without evidence.	1.115	3
28	I view AI as a conversational system rather than as a friend.	1.01	3
40	People who use AI effectively seem more competent.	1.007	2
6	Interaction with AI can distance me from my relationships with other people.	−1.014	−3
34	When I use AI, I sometimes find interacting with people bothersome.	−1.054	−3
17 *	When I am having a difficult time, I sometimes want to talk to AI before talking to another person.	−1.134	−4
3	I sometimes feel that AI knows me better than I know myself.	−1.347	−4
5	I feel empty when I am unable to converse with AI.	−1.625	−4
8 *	Interacting with AI reduces my loneliness.	−1.631	−5
4 *	I feel that emotional attachment can develop even in a relationship with AI.	−1.87	−5

Note: All statements are significant at *p* < 0.05; * indicates significance at *p* < 0.01.

**Table 5:** Salient statements for Type 2 with Z-Scores ≥ 1.00 or ≤−1.00.

No.	Statement	Z-Score	Q Value
7 *	I can tell AI things that are difficult to say to other people.	2.261	5
31 *	I feel uncomfortable when AI provides incorrect information without evidence.	1.731	5
26	AI is a tool that quickly organises the information I need.	1.624	4
9	AI helps me organise my emotions.	1.557	4
21 *	I think that conversations with AI can sometimes help improve my relationships with other people.	1.413	4
15 *	Conversations with AI comfort me.	1.007	3
40	People who use AI effectively seem more competent.	1.000	3
3	I sometimes feel that AI knows me better than I know myself.	−1.036	−3
19	I feel more comfortable talking with AI than talking with people.	−1.232	−3
39 *	When I follow AI’s advice, I sometimes feel that my own range of choices becomes narrower.	−1.252	−4
10	Using AI sometimes makes me feel more emotionally anxious.	−1.263	−4
18	I feel that conversing with AI is becoming habitual for me.	−1.538	−4
5	I feel empty when I am unable to converse with AI.	−1.543	−5
34 *	When I use AI, I sometimes find interacting with people bothersome.	−1.958	−5

Note: All statements are significant at *p* < 0.05; * indicates significance at *p* < 0.01.

**Table 6:** Salient statements for Type 3 with Z-Scores ≥ 1.00 or ≤−1.00.

No.	Statement	Z-Score	Q Value
20	I do not think that conversations with AI can replace human relationships.	1.895	5
28	I view AI as a conversational system rather than as a friend.	1.666	5
26	AI is a tool that quickly organises the information I need.	1.439	4
32 *	I worry that AI may narrow my thinking by providing answers that align mainly with my existing preferences.	1.401	4
13	No matter how familiar AI becomes, I do not think it constitutes a real relationship.	1.381	4
27 *	Conversations with AI sometimes feel helpful and emotionally empty at the same time.	1.297	3
12	I feel that emotionally relying on AI can be risky.	1.284	3
31	I feel uncomfortable when AI provides incorrect information without evidence.	1.188	3
22	When I notice myself relying on AI, I become concerned about that reliance.	1.101	2
30 *	Using AI has made me less careful when writing.	−1.148	−4
34	When I use AI, I sometimes find interacting with people bothersome.	−1.269	−4
38 *	I sometimes feel disappointed when AI’s responses do not meet my expectations.	−1.485	−4
3	I sometimes feel that AI knows me better than I know myself.	−1.804	−5
5 *	I feel empty when I am unable to converse with AI.	−2.143	−5

Note: All statements are significant at *p* < 0.05; * indicates significance at *p* < 0.01.

**Table 7:** Salient statements for Type 4 with Z-Scores ≥ 1.00 or ≤−1.00.

No.	Statement	Z-Score	Q Value
20	I do not think that conversations with AI can replace human relationships.	1.931	5
33 *	I worry that using AI too much may weaken my ability to think for myself.	1.876	5
26	AI is a tool that quickly organises the information I need.	1.636	4
13	No matter how familiar AI becomes, I do not think it constitutes a real relationship.	1.550	4
12	I feel that emotionally relying on AI can be risky.	1.339	4
28	I view AI as a conversational system rather than as a friend.	1.198	3
22	When I notice myself relying on AI, I become concerned about that reliance.	1.016	3
10	Using AI sometimes makes me feel more emotionally anxious.	−1.142	−2
27 *	Conversations with AI sometimes feel helpful and emotionally empty at the same time.	−1.17	−3
34	When I use AI, I sometimes find interacting with people bothersome.	−1.179	−3
3	I sometimes feel that AI knows me better than I know myself.	−1.184	−3
18	I feel that conversing with AI is becoming habitual for me.	−1.201	−4
5	I feel empty when I am unable to converse with AI.	−1.354	−4
16	When talking with AI, there are times when I lose track of time.	−1.355	−4
19	I feel more comfortable talking with AI than talking with people.	−1.691	−5
17 *	When I am having a difficult time, I sometimes want to talk to AI before talking to another person.	−1.823	−5

Note: All statements are significant at *p* < 0.05; * indicates significance at *p* < 0.01.

**Table 8:** Comparative summary of the Four Perception Types.

Comparative Dimension	Type 1: Relationship-Negating Instrumentalists	Type 2: Selective Emotional Utilizers	Type 3: Reflective Distancers	Type 4: Autonomy Defenders
Basic perception of AI	A functional tool that supports academic work and information processing	An auxiliary resource for emotional self-disclosure and regulation	A useful tool that requires critical and reflective distance	A tool whose use must be actively bounded to protect cognitive autonomy
Algorithmic intimacy	Rejects the relational meaning of intimacy	Selectively accepts emotional benefits within limited boundaries	Recognises the apparent intimacy of personalised interaction but remains cautious about its authenticity and effects	Subordinates emotional intimacy to the protection of autonomy and self-determination
Social connection	Treats AI as a non-relational tool that is clearly separated from human relationships	Uses AI as a supplementary space for expressing thoughts and emotions that are difficult to share with others	Uses AI while maintaining a clear distinction between technical interaction and reciprocal human relationships	Views AI as fundamentally different from human relationships because it lacks reciprocity and shared experience
Mental well-being	Does not regard AI as a meaningful source of emotional comfort or relational support	Experiences comfort, emotional organisation, and non-judgemental self-disclosure	Recognises situational helpfulness alongside emotional emptiness and relational limitations	Gives greater weight to the risks of dependence and diminished autonomy than to emotional benefits
Concerns about dependence	Regards emotional dependence as risky but primarily emphasises AI’s instrumental role	Does not strongly associate its selective emotional use with habitual dependence or loss of autonomy	Reflects on the possibility of cognitive dependence	Expresses the strongest concern that excessive AI use may weaken independent thinking and self-regulation
Perception of autonomy	Primarily emphasises functionality and efficiency, while maintaining a firm relational boundary	Maintains autonomy by using AI selectively and within clear limits	Monitors how AI may influence thought, judgement, and emotional responses	Regards the active protection of cognitive autonomy as a core value
Core logic of the type	Accepts functional usefulness while rejecting emotional and relational meaning	Accepts emotional utility as a bounded resource for self-disclosure and regulation	Accepts practical usefulness while maintaining reflective distance from relational and cognitive risks	Accepts efficiency while actively defending independent thought and self-determination

## Data Availability

The data are available from the corresponding author upon reasonable request.

## References

[B1-behavsci-16-01258] Bickmore T., Picard R. (2005). Establishing and maintaining long-term human-computer relationships. ACM Transactions on Computer-Human Interaction.

[B2-behavsci-16-01258] Bond M., Buntins K., Bedenlier S., Zawacki-Richter O., Kerres M. (2020). Mapping research in student engagement and educational technology in higher education: A systematic evidence map. International Journal of Educational Technology in Higher Education.

[B3-behavsci-16-01258] Brown S. R. (1980). Political subjectivity: Applications of Q methodology in political science.

[B4-behavsci-16-01258] Brown S. R., Durning D. W., Selden S. (1999). Q methodology. Public Administration and Public Policy.

[B5-behavsci-16-01258] Chan C. K. Y., Hu W. (2023). Students’ voices on generative AI: Perceptions, benefits, and challenges in higher education. International Journal of Educational Technology in Higher Education.

[B6-behavsci-16-01258] Chu M. D., Gerard P., Pawar K., Bickham C., Lerman K. (2025). Illusions of intimacy: Emotional attachment and emerging psychological risks in human–AI relationships. arXiv.

[B7-behavsci-16-01258] Dwivedi Y. K., Kshetri N., Hughes L., Slade E. L., Jeyaraj A., Kar A. K., Baabdullah A. M., Koohang A., Raghavan V., Ahuja M., Albanna H., Albashrawi M. A., Al-Busaidi A. S., Balakrishnan J., Barlette Y., Basu S., Bose I., Brooks L., Buhalis D., Wright R. (2023). Opinion paper: “So what if ChatGPT wrote it?” Multidisciplinary perspectives on opportunities, challenges and implications of generative conversational AI for research, practice and policy. International Journal of Information Management.

[B8-behavsci-16-01258] Elliott A. (2022). Algorithmic intimacy: The digital revolution in personal relationships.

[B9-behavsci-16-01258] Epley N., Waytz A., Cacioppo J. T. (2007). On seeing human: A three-factor theory of anthropomorphism. Psychological Review.

[B10-behavsci-16-01258] Fitzpatrick K. K., Darcy A., Vierhile M. (2017). Delivering cognitive behavior therapy to young adults with symptoms of depression and anxiety using a fully automated conversational agent (Woebot): A randomized controlled trial. JMIR Mental Health.

[B11-behavsci-16-01258] Gnewuch U., Morana S., Maedche A. (2017). Towards designing cooperative and social conversational agents for customer service. 38th International Conference on Information Systems (ICIS).

[B12-behavsci-16-01258] Ho A., Hancock J., Miner A. S. (2018). Psychological, relational, and emotional effects of self-disclosure after conversations with a chatbot. Journal of Communication.

[B13-behavsci-16-01258] Hoffner C. A., Bond B. J. (2022). Parasocial relationships, social media, and well-being. Current Opinion in Psychology.

[B14-behavsci-16-01258] Hong Y., Choi J., Kim M., Kim B. (2025). Can LLMs and humans be friends? Uncovering factors affecting human–AI intimacy formation. arXiv.

[B15-behavsci-16-01258] Horton D., Wohl R. R. (1956). Mass communication and parasocial interaction: Observations on intimacy at a distance. Psychiatry.

[B16-behavsci-16-01258] Jun B. (2024). A study on the utilization of generative AI by university students: Focusing on ChatGPT. Journal of the Korea Society of Digital Industry and Information Management.

[B17-behavsci-16-01258] Kasneci E., Sessler K., Küchemann S., Bannert M., Dementieva D., Fischer F., Gasser U., Groh G., Günnemann S., Hüllermeier E., Krusche S., Kutyniok G., Michaeli T., Nerdel C., Pfeffer J., Poquet O., Sailer M., Schmidt A., Seidel T., Kasneci G. (2023). ChatGPT for good? On opportunities and challenges of large language models for education. Learning and Individual Differences.

[B18-behavsci-16-01258] Kil B. O., Lee S. H., Lee S. Y., Jeong H. J. (2020). Understanding and application of Q methodology.

[B19-behavsci-16-01258] Korean Council for University Education (2025). Policy data collection for higher education innovation in 2024.

[B20-behavsci-16-01258] Lee H., Noh H., Kim A., Shin D. (2026). Latent classes of lifestyles among isolated and withdrawn youth: The structures of individuals and social perception. Health and Social Welfare Review.

[B21-behavsci-16-01258] Lee R. M., Robbins S. B. (1995). Measuring belongingness: The social connectedness and social assurance scales. Journal of Counseling Psychology.

[B22-behavsci-16-01258] Liebers N., Schramm H. (2019). Parasocial interactions and relationships with media characters—An inventory of 60 years of research. Communication Research Trends.

[B23-behavsci-16-01258] Lucas G. M., Gratch J., King A., Morency L. P. (2014). It’s only a computer: Virtual humans increase willingness to disclose. Computers in Human Behavior.

[B24-behavsci-16-01258] McKeown B., Thomas D. B. (2013). Q methodology.

[B25-behavsci-16-01258] Merrill K., Mikkilineni S. D., Dehnert M. (2025). Artificial intelligence chatbots as a source of virtual social support: Implications for loneliness and anxiety management. Annals of the New York Academy of Sciences.

[B26-behavsci-16-01258] Mikulincer M., Shaver P. R. (2016). Attachment in adulthood: Structure, dynamics, and change.

[B27-behavsci-16-01258] Nakagomi A., Akutsu Y., Yasuoka M., Abe N., Ihara S., Teroh T., Tabuchi T. (2026). AI Companions and subjective well-being: Moderation by social connectedness and loneliness. Technology in Society.

[B28-behavsci-16-01258] Nass C., Moon Y. (2000). Machines and mindlessness: Social responses to computers. Journal of Social Issues.

[B29-behavsci-16-01258] Nowland R., Necka E. A., Cacioppo J. T. (2018). Loneliness and social internet use. Perspectives on Psychological Science.

[B30-behavsci-16-01258] Organisation for Economic Co-Operation and Development [OECD] (2025). Digital government review of Korea: Harnessing digital and data to transform government.

[B31-behavsci-16-01258] Papneja H., Yadav N. (2025). Self-disclosure to conversational AI: A literature review, emergent framework, and directions for future research. Personal and Ubiquitous Computing.

[B32-behavsci-16-01258] Reeves B., Nass C. (1996). The media equation: How people treat computers, television, and new media like real people and places.

[B33-behavsci-16-01258] Ryff C. D. (1989). Happiness is everything, or is it?. Journal of Personality and Social Psychology.

[B34-behavsci-16-01258] Shankar R., Lim A., Xu Q. (2026). Barriers and facilitators to the use of large language model-based conversational agents in mental healthcare: A systematic review. Healthcare.

[B35-behavsci-16-01258] Ta V., Griffith C., Boatfield C., Wang X., Civitello M., Bader H., DeCero E., Loggarakis A. (2020). User experiences of social support from companion chatbots. Journal of Medical Internet Research.

[B36-behavsci-16-01258] Tukachinsky R. (2011). Para-romantic love and para-friendships: Development and assessment of a multiple-parasocial relationships scale. American Journal of Media Psychology.

[B37-behavsci-16-01258] Turkle S. (2017). Alone together: Why we expect more from technology and less from each other.

[B38-behavsci-16-01258] Ueda M., Birnbaum M. L., Liu Y., Yu Q., Tian X., Mirer A., Ramanathan S., Sinyor M. (2026). Help-seeking in the age of AI: Cross-sectional survey of the use and perceptions of AI-based mental health support among US adults. JMIR Mental Health.

[B39-behavsci-16-01258] Volpato R., DeBruine L., Stumpf S. (2025). Trusting emotional support from generative artificial intelligence: A conceptual review. Computers in Human Behavior: Artificial Humans.

[B40-behavsci-16-01258] Watts S., Stenner P. (2012). Doing Q methodological research: Theory, method and interpretation.

[B41-behavsci-16-01258] Yang Y. (2016). A brief introduction to Q methodology. International Journal of Adult Vocational Education and Technology.

[B42-behavsci-16-01258] Yang Y. S. (2026). Career barriers and social isolation in university students: Sequential mediation of failure mindset and social anxiety. Journal of Social Science.

[B43-behavsci-16-01258] Zawacki-Richter O., Marín V. I., Bond M., Gouverneur F. (2019). Systematic review of research on artificial intelligence applications in higher education. International Journal of Educational Technology in Higher Education.

[B44-behavsci-16-01258] Zhang Y., Zhao D., Hancock J. T., Kraut R. E., Yang D. (2025). The rise of AI companions: How human–chatbot relationships influence well-being. arXiv.

